# A qualitative study of what care workers do to provide patient safety at home through telecare

**DOI:** 10.1186/s12913-021-06556-4

**Published:** 2021-06-05

**Authors:** Randi Stokke, Line Melby, Jørn Isaksen, Aud Obstfelder, Hege Andreassen

**Affiliations:** 1 Centre for Care Research, NTNU Norwegian University of Science and Technology, P.O. Box 191, 2802 Gjøvik, Norway; 2 Faculty of Social and Health Sciences, Inland Norway University of Applied Sciences, Gudbrandsdalsvegen 350, 2624 Lillehammer, Norway; 3 Centre for Women and Gender Research, UiT, The Arctic University of Norway, P.O Box 6050 Langnes, 9037 Tromsø, Norway

**Keywords:** Care work, Home care practice, Safety work, Invisible work, Social alarm, Telecare, Welfare technology

## Abstract

**Background:**

In health care, the work of keeping the patient safe and reducing the risk of harm is defined as *safety work*. In our digitised and technology-rich era, safety work usually involves a relationship between people and technologies. Telecare is one of the fastest-growing technology-domains in western health care systems. In the marketing of telecare, the expectation is that safety is implicit simply by the presence of technology in patients’ homes. Whilst both researchers and health authorities are concerned with developing cost-benefit analyses and measuring effects, there is a lack of attention to the daily work needed to ensure that technologies contribute to patient safety.

This paper aims to describe *how* patient safety in home care is addressed through and with telecare. We base our exploration on the social alarm, an established technology that care workers are expected to handle as an integrated part of their ordinary work.

**Methods:**

The study has a qualitative explorative design where we draw on empirical data from three case studies, involving five Norwegian municipalities that use social alarm systems in home care services. We analyse observations of practice and interviews with the actors involved, following King’s outline of template analysis.

**Results:**

We identified three co-existing work processes that contributed to patient safety: “Aligning people and technologies”; “Being alert and staying calm”; and “Coordinating activities based on people and technology”. Attention to these work processes exposes safety practices, and how safety is constructed in relational practices involving multiple people and technologies.

**Conclusions:**

We conclude that the three work processes identified are essential if the safety alarm is to function for the end user’s safety. The safety of home-dwelling patients is reliant on the person-technology interface. The efforts of care workers and their interface with technology are a central feature of creating safety in a patient’s home, and in doing so, they utilise a repertoire of skills and knowledge.

## Background

### Working to maintain patient safety

The focus on patient safety is a movement within health and care services that arose when the Institute of Medicine in the US published the report *To err is human: building a safer health system* [1]. Since then, although the focus on patient safety has gained increased interest around the world, this is mainly within a hospital setting even though most patients are in primary care, including home care services. There is, therefore, a need for more knowledge about the safety work performed in home care, especially in light of the care services becoming increasingly complex and thereby involving more risks for the patients [2–4].

To address this gap, in this paper we draw on sociological insights on healthcare work and organisation, to explore the work performed to ensure patient safety in home care services in depth.

In their pivotal studies, Strauss et al. [5] point to care workers’ engagement in different kinds of work activities, and different types are outlined. They stress that organising work is a major part of the span of healthcare duties. In doing so, they were among the first to challenge the opinion that care work is solely clinical work at the bedside. Other scholars, for example, Davina Allen [6] have followed this line of argumentation, emphasising that ‘organising work’ is a vital part of care work, and consequently for positive patient outcome and experiences.

Another work type described is safety work that – not surprisingly – pertains to creating patient safe environments. This includes not only efforts to protect patients from the safety risks posed by the illnesses but also risks posed by the environment, the relevant technology, the service, and the organisation in which the service takes place. Concerning the technology involved, this safety work focuses on the purchase and installation of the technology, ongoing maintenance of the technology within the context of use, and the person technology interface. Due to the complexity of the environment and care needs, there are many unknown risks related to technology use.

However, other kinds of activities (work types) are also highly relevant to understand care workers’ efforts in establishing patient safety. One important type of work is m*achine work,* e.g. the practical work with technologies such as syringe pumps, ventilators, and blood pressure monitors in addition to digital technologies like different telecare solutions. Traditionally, such work was mostly reserved for care workers in hospitals, but today machine work is becoming widespread within home care. The dramatically increasing use of technologies has a wide-ranging impact on the delivery and organisation of healthcare, on both professional practice and patients’/families experience of illness and its management, including the feeling of safety. Different terms can be used to reference these technological devices and this paper use the umbrella term “telecare.” “Telecare” is covering different technologies that are expected to contribute to maintaining or improving individuals’ functioning, safety, and independence, often in a home care setting. Other terms partly overlapping are “welfare technologies,” “telehealth,” “ambient assisted living technologies,” “telemedicine,” and “e-health.” Although these terms all cover different forms of digital care, there is considerable overlap among them [7, 8].

Yet another work type is *articulation work*. This is sometimes popularly referred to as ‘work that makes the work work’. In practice, this means all sorts of connection, integration, coordination and organisational efforts conducted so that the patient trajectory evolves in the desired direction [5].

To summarise, home care workers engage in a range of different work types to facilitate patient safety. These work types are performed simultaneously and intertwined in the daily work of the care workers. In addition to the various forms of work performed by healthcare professionals, we should not forget all the work that must be done by patients/family/friend and other carers during the course of a caring trajectory [9]. Applying this broad perspective on care work enables us to see the whole range of care workers’ (from registered nurses to unskilled health care workers) activities in the pursuit of establishing the conditions that foster a safe environment for patients at home, including a different kind of telecare technologies.

To add to the complexity of different kinds of work and technologies, care work takes place in complex services characterised by ‘wicked problems. Wicked problems are complex problems characterised by disagreements regarding the nature of the problem, different and changing stakeholders, competing interests, rules and regulations, part-time workers and other work issues [10]. An example of this is described in a Norwegian study of safety work in nursing homes and home care. They found that major threats related to patient safety are the heavy workload and the lack of competence among the personnel [11].

To sum up, the research describes that work in the intersection of technologies and healthcare is hard to plan, and requires actors to make continuous adjustments to their plans [6, 12, 13].

### Patient safety through technology

Policymakers and healthcare providers are increasingly suggesting telecare as a solution to promote patient safety in patients’ homes. We are not the first to point out the shortage of studies on what it takes for telecare to promote patient safety in home care [3]. To date, the research in this field has been dominated by effectiveness studies, an approach that diverts our attention from the effort invested by those involved in the care to make telecare work in the first place [6]. Telecare is often promoted as an easy fix to the increasing pressure to deliver high-quality services to ever more patients with ever greater needs [8, 14]. Previous research on ‘technology in use’ in other parts of health care shows that gainful implementation of technology in work practices must handle the intricate and rather unpredictable relationships between people and technologies [9, 15, 16].

### The social alarm

To explore the efforts of care workers to foster patient safety through telecare, we have focused on the social alarm as a typical example of telecare used to safeguard frail people living independently at home. The social alarm is a technological device consisting of a unit placed centrally in the home and a pendant, a necklace/wristband with a button that the user can press when in need. Pressing the button allows open communication with a dedicated responder, enabling proper response (normally from the home care nurse) [17–19], and is often the starting point for additional applications. The social alarm is frequently described as a “plug and play” device and has been in use in health care services throughout most western countries for 40 years [13, 20]. Today, social alarms are widely adopted in home care services, and routinely embedded in service delivery and patients’ everyday lives [18]. Thus, the devices can be considered as ‘normalised’ technologies [21], suggesting that they are easy to use and that the desired effects are easily accomplished [22]. However, there are still issues related to how the alarms are used by the patients, their functions and the organisation of their use within local services providers. For example, the main unit must be carefully installed in the alarm user’s home, and the alarm receiver must be able to communicate when the alarm is activated. The patient must know the range of the pendant both inside and outside the house as this varies depending on the building structure and materials. The social alarm is usually organised as an integrated part of the home care services, entailing responsibility for responding to the alarm when activated. Furthermore, as the newer social alarms have the potential to incorporate a range of functionalities, such as fall alarm devices, blood pressure devices and gas and smoke detectors [17, 18, 23], the complexity of the interplay between the care workers, patients and the technical specificities of the social alarm, have increased considerably.

In previous studies, the first author has discussed how social alarms paradoxically both contribute to safety and give rise to safety risks for the patient, and that the social alarm needs a lot of work and adjustments to function in a way that contributes to safety [24]. Other scholars have shown that care workers play an important role in making the social alarm easy to use for patients through ongoing information and support for the users [25, 26]. This line of research illustrates how the ‘ease of use’ and the ‘normalised’ social alarm seems to be the outcome of intricate connections between people and technology in everyday care work, resting on the interplay of care workers, patients, and the technical specificities of the social alarm, such as whether they rely on analogue or digital systems.

### Technology and care work – two sides of the same coin

Care work is not just based on pure human relationships, but shaped by continuous interconnections of devices, tools, routines, symbols, knowledge, rules and people, which must continuously be maintained [27, 28]. The smallest changes in the technologies, the people involved, or the organisation of the service have the potential to impact the intricate network of people and technologies that care work is made up of.

We may say that constructing patient safety in care work is the outcome of how technologies and people are intertwined in practical tasks related to the provision of services for patients. The actors involved in the practices – be it technologies or people - emerge as part of a web of relationships and mutual dependencies to which they contribute [29]. Thus, the technologies are not ‘things’ with a specific feature that have particular effects when implemented correctly, but specific practices that are inherently contingent, unfolding in specific places, time and contexts.

In this paper, we highlight how these complex relations between telecare and care workers’ activities play out in daily practice by asking what the care workers do to make telecare function as a tool for patient safety. Our research is based on an empirical study in five Norwegian municipalities where we employ theoretical perspectives of technology in practice, i.e., perspectives investigating the dialectic relationship between technology and its users in health care (Timmermans and Berg, 2003). Through a discussion drawing on such contributions from Pols, Greenhalgh and Johanessen [9, 15, 16]. By doing so, we aim to provide insight into what this safety work implies and how it is performed in day-to-day care work.

## Methods

This paper aims to describe and analyse the complex relations between telecare and safety work in home care services. Through a qualitative explorative design, we aim to contribute with knowledge about the details of daily care work in technology-supported home care services. Using a variety of qualitative methods opens up for larger breadth and depth in the data material, and enabled a comprehensive picture of practice [30, 31]. By analysing in-depth individual and focus-group interviews and observational data we are exploring how care workers engage and work with and around the social alarm to achieve safety and independent living for their patients.

### Methods for data collection

The empirical material was originally collected in three separate projects involving five Norwegian municipalities that all investigated the implementation of social alarms as presented in Table 1 [19, 26, 32, 33]. The data material was joined in one corpus of data for this study and all the data material is included in the analysis of this study. Data were collected in a combination of participant observation, individual interviews and focus group interviews following a qualitative study design. This gave the researcher insights into how the care workers facilitated patient safety when working with the social alarm.

**Table 1:** The three cases included in the study

The three cases		Methods	Characteristics of interview participants
Case 1	Two municipalities	Individual interviews 2-week participant observations	6 care workers, two of whom are key workers responsible for the social alarm
Case 2	Two municipalities	Focus group interviews.	17 care workers and 1 technician.
Case 3	One municipality	Focus group interviews and individual interviews	12 care workers and 2 management workers

The municipalities were strategically chosen for maximum diversity, as they represented both typical and very different municipalities in Norway concerning the type of alarm, ways of organising the social alarm, size and geography as presented in Table 2. All included municipalities had long time experience with the social alarm.

**Table 2:** Describes the organisation, size and geography of included municipalities

Municipality	Integration and organisation of the social alarm	Size and geography
1	The municipality was changing to digital alarms. When the alarm was activated there was a direct connection to home care workers	Mid-size city in the Inland region with 30,000 inhabitants.
2	They had both analogue and digital alarms. A private call centre received alarm calls and allocated them further to home care services	Rural location on an island in the north of Norway with 2600 inhabitants.
3	They had analogue alarms and were trying out digital alarms with added devices. The alarms calls were received at a public call centre and allocated further to home care services.	Mid-size rural location in the Inland with 8000 inhabitants
4	They had analogue alarms and were trying out digital alarms with added devices to new user groups. The alarms calls were received at a public call centre and allocated further to home care services.	Mid-size rural location in the Inland with 13,000 inhabitants
5	They had an analogue alarm system and were trying out a digital new system.	Rural location in the mountains with less than 2000 inhabitants

In all five municipalities, social alarm systems for home-dwelling patients had already been implemented prior to our research.

The managers of the home care service in each municipality recruited participants for the interviews. They had all good knowledge of the home nursing service; how it was organised, its routines and patient population. The informants had positions as leaders, registered nurses, and assistant nurses. An inclusion criterion was that they had experience with the social alarm over time. The interviews were conducted at the care workers work locations. Due to anonymity issues, the profession of care workers is not described. The two-week full-time observation in case one was conducted in the home care services there the researcher was following the home care workers in all their work related to the social alarm. Regarding the focus groups interviews in case two and three, there were 5–7 informants. The informants were chosen by the head of department and head of services who also participated in the interviews as informants. Some of the informants had been involved in preparing the department for the shift in the alarm system; form an analogue alarm system to a digital alarm system, and others had responsibilities for supporting their colleagues in the use of the new digital alarms.

The interviews lasted between 30 min and two hours and were organised as informal conversations where the care workers individually or in groups were invited to share their experience on the social alarms. The participants described comprehensive stories, and this provided a rich material.

### Analytical approach

To ensure a systematic and valid analysis we built on King’s outline of template analysis [34] and thus followed an inductive-deductive approach. Template analysis is a practically oriented, iterative thematic approach to a topic from differing perspectives, and can be used within different research traditions [34].

Through the template analysis we conducted the following steps:

First, an initial template, based on one transcript from each case, was developed. From this, a coding scheme that set out four a priori (tentative) themes were created. The themes were identified through the initial analysis of the three cases as presented in Table 3.

**Table 3:** Tentative advanced themes

Tentative themes:	
Articulation of the alarm work. How is the alarm work articulated by the care worker?	
Alarm work as additional work.	
When telecare functionalities collide with established routines.	
Emotions and sense-making - Expectations, pressure, uncertainty and other aspects of work.	

The coding scheme opened up the data for further analysis of the entire empirical material, allowing for “modifying and revising the initial template” [34] through an exploration of the data and discussions in the research group. This provided a synthesis of data from the transcripts within the different codes, allowing for a hierarchical organisation of codes, where groups of similar codes were clustered together to produce more general, higher-order codes. This also involved a critical validation of the interpretation within the research group. We then identified the main themes from the codes and identified and discussed relationships among themes. Quotes to illustrate the themes where identified.

The analysis identified three main work processes/categories that study participants engaged in to optimize the contribution of the social alarm to safety and independent living for the users: “Aligning people and technologies”, “Being alert and staying calm” and “Coordinating activities depending on people and technology”**.** Below we describe the detailed work practices involved in the three processes.

## Results

The home care workers created a chronological story of the technology in use, by describing how they worked with the social alarm to ensure safety for the patients.

### Aligning people and technologies

In all the municipalities included in the study, the patient applied to the municipality for the social alarm when they felt in need of the service. The care workers then performed a formal assessment of whether the social alarm was a suitable technology. Aligning users and alarms can be considered the first step in creating safety for people who live at home:

*Selecting patients is thus not a straightforward process but triggered thoughts and reflections on which patients should have a social alarm and what kind of alarm they should have.*

Providing patients with the right kind of devices while considering patient safety was not straightforward and could be demanding in terms of expertise and time. The choice of the right technologies was characterised by negotiations between healthcare workers, patients and families. It was necessary to align their wishes, ability to cope and needs with technologies to address patient safety. Additionally, the care workers also needed experience with and knowledge of relevant technology. This could be a burden for the care workers, as we can see from the next quote:

*"We cannot familiarise ourselves with everything. Which alarm is most suited for that patient? We know what they need, but it’s not easy for us to find the right type. (…) And maybe you also must try out different types. And you must monitor what happens. There are constantly new technologies being introduced, so following the development…. [is time-consuming].*”

To avoid a mismatch between a patient and the technology, the care workers needed to draw on several aspects of their professional competence and carry out different types of tasks.

#### Setting up the alarm: getting it to function

The next step was to set up the alarm in the patient’s home. This included adjusting the alarm to the patient’s environment and the home care services’ work routines. Study participants described the work of setting up the social alarm in the patient’s home as comprehensive as described in the quote below. Furthermore, after setting it up, they had to ensure that the social alarm was functioning. The quote below illustrates the wide range of tasks involved in this work-process:

*I have the responsibility for all decisions on new social alarms. That means I must find a suitable alarm, send a form to the alarm centre, and install the alarm. Sometimes I try to delegate the task. I have tried to show others how to do it (… ) I buy a SIM card and fix it. We have decided that the municipality will take care of this. If it was left to the individual patient, it would delay the process and it would be less transparent (…). And if there are errors in relation to the alarms, I will always be kept informed – they just write messages to me describing the error. But there are over a hundred alarms now, and there are errors regularly. So now I have said [to the patient] that you must bring the alarm to the alarm centre Monday or Thursday or call them. Because I cannot repair an alarm. (…) And it’s my job to deactivate social alarms. If somebody dies or moves, I will send in a form and the alarm will be deactivated. I might also go to their home and fetch the alarm, or I will call their next of kin and ask them to deliver it to us*.

The care worker’s story illustrates the wide range of tasks needed to get the social alarm functioning, and thereby contribute to patient safety. Patient safety is achieved by combining procedures, administrative skills, and collaboration with home care recipients and their families. Collaboration between home care workers and technical support is also crucial to the alarm system infrastructure and integrity. Our analysis revealed that there were requirements concerning the delivery and organisation of safety work/patient safety that involved multiple actors that needed to be aligned. Some collaborated with technology vendors; others with another department within their municipality, or both. The technical service department or fire department were often involved because they have round-the-clock services and technical staff. An informant with special responsibility for the social alarm described how difficult it was to collaborate with others regarding this due to different work hours. Moreover, it was often unclear whose responsibility it was when something went wrong. Even though the technical work with the alarm was straightforward, sometimes things became challenging and technical skills fell short:

*..I had many conversations with him (the technician) when I went out to connect an alarm. I called him and said: ‘I am standing here: where should I put the wires?’ Yes, and then he remembered exactly what the alarm looks like underneath, what connections there are... and what the different contacts looked like, so he explained the process to me: First you put the white wire in the middle hole of the alarm device… And that is where I got guidance.*

The social alarm function was dependent on practical negotiations and collaboration between multiple actors. These practices are complex in the sense that home care workers must draw on different kinds of knowledge and a wide range of other people as well as practical skills to complete tasks. Furthermore, these tasks require time and attention and they are added on top of the care professionals’ traditional ‘care tasks. Even though the technology is an integrated part of usual care it seems that its inclusion is lagging as it is not included in the planning of the work schedule of the care workers.

### Responsibility for the alarm: “Being alert and staying calm”

When a user pressed the alarm, a signal was transferred to a mobile phone. On each shift, individual care workers were given the responsibility for the ‘alarm phone’. The care workers responsible for managing the alarms on a shift were not exempted from other care tasks, and they handled the ‘alarm phones’ in addition to these.

The care workers described how the alarm calls had priority over other forms of care work as the alarm calls often involved possible life-threatening situations:

*.. then we must go there at once because the person might have fallen and injured themselves.. or.. they might even die...*

Even though many of the informants described how the social alarm was always prioritised, one care worker described how they sometimes ignored the alarm for a short time when busy with another patient. She further described how this felt wrong. Whatever choice the care workers made regarding priorities in these situations, it had direct consequences for patient safety, both for the patient who had triggered the alarm and for the patient who was left behind.

The care workers described hectic workdays where they had lists of patients they had to attend to and help in their home. Work in connection with the social alarm was prioritised overall home care work. A care worker involved with another patient when the alarm was activated, interrupted the interaction and care of the patient to answer the alarm call due to client confidentiality. This was disturbing for both the care worker and the patient. The person receiving care in their home could be in a vulnerable situation, perhaps with parts of their body exposed for wound cleaning, bathing, or other care procedures. Care workers thus had to prioritise between giving attention to different patients and their safety. Sometimes the patients whom the care worker was visiting at the time of the alarm commented on these interruptions. However, in general, they sympathised with the demands imposed on the care worker.

*Some (talking about the patient) are very understanding. They’re used to us going in and out of situations. They feel sorry for us when we have to leave the room all the time.*

The care workers never knew if, when and how often the alarm would be activated during a shift, and they never knew what they would be exposed to during this work, or how time-consuming it would be. This kept them constantly alert and prepared for dramatic events. They explained that this led to stress, as they had to answer the alarm and be with another patient at the same time. The informants reported that collaborators and colleagues who never answered the alarm did not understand the responsibility of having the alarm phone.

Informant: *I don’t think people are aware of what it involves for us, maybe. We do have a great responsibility. We do have (the alarm) all the time when we are at work.*

Informant: *You never know what will happen.*

In two of the cases in this study, patients had mobile social alarms, allowing them to use the alarm away from the home. This opened for new freedom for the patients, but also further extended care workers’ responsibility for the patients’ safety and adding to the unpredictability of the work.

Informant: *Is it so that they can take it (the alarm) with them to the shop? Are we supposed to come if the alarm is activated? That is a tremendous responsibility for us.*

The mobile alarm enabled users to travel to their holiday home or elsewhere, and still be able to activate the alarm. This forced the service to think ahead and consider what services to offer the service users. The service is extended so the patient can stay more active, but care workers’ responsibilities increased even more. It might be difficult to find the patient when the alarm was activated.

Informant: *And everybody says: So, you have a social alarm, that’s nice. But they don’t know what it implies. They know it provides safety for our patients, but they don’t know what lies behind it in relation to the emotions, feeling of responsibility, bad conscience etc. for those in the care service.*

Being responsible for the social alarm entails a professional assessment of both the needs related to the alarm and insights into what the activation of an alarm implies. Furthermore, the effectiveness of the alarms as technological products depends on their integration into other organisational arrangements to provide around-the-clock service. This work is further described below.

### Coordinating activities depending on people and technology

To succeed with social alarms, care workers had to continually adjust procedures and were involved in negotiations, giving guidance, tinkering and working out procedures. In short, they had to put in place a structure that the patients could utilise when in need.

Technological failure caused some of the informants to wonder whether they could rely on technology. They described how patients sometimes worried about whether the alarm worked, especially when they struggled to make it function.

*“Daring to rely on technology. Daring to rely on sensors, handling the reception of alarms. Here ... there are quite a few changes.”*

Study participants also described how they worried about the patients and were afraid that the technology would not work when needed, particularly when mobile phone signals in the area were unstable. This made them worry about whether to trust the system:

*It was stressful, never knowing whether the alarm system worked. When you discovered that the system was malfunctioning, you wondered: Who is lying out there now. Who has activated the alarm?*

Competencies, or the lack of competencies, was evident as interviews revealed different descriptions of how the care workers needed know-how knowledge, not just knowing what to do and when to do it, but also what to do when needed. These competencies require special training and education, depending on the context in which the competencies will be used.

Another practice born out of the technologies was the delegation of responsibility towards the care workers, which the previous quote also touches upon. This is explicit in the following citation where one municipality tried out a new system, and the care worker describes what changes they experienced when they implemented new alarms:

*Informant: “[the new system] increases the users’ feeling of being kept safe when they know that they hear us loud and clear and that we respond quickly. It also increases the feeling of security for the care workers when several of us are logged on to [the alarm system]. This functionality allows us to tell the system that we are busy, and then the alarm will be delegated to the next care worker. On the old system we had two alarm telephones and [on this system we could not report as busy]. Now [with the new system] we hear them when they [the users] press the alarm. Often, they just want to ask a question. Previously we would have made a visit to determine the cause of the alarm. […] There’s a sense of security in the fact that you know, if you are busy, the alarm will go to another care worker, or it will return to you.”*

This quote underlines how the new system in this one municipality brings a sense of safety to both user and care worker, through the functionality of responsibility delegation. We can extrapolate from the quote that care workers’ responsibility is increasing, but at the same time, they can resolve situations faster. By not being constantly interrupted by alarm calls, the care workers can (in theory at least) work more efficiently on their current task.

Most of the informants’ reported challenges were linked to establishing good practices around the use of social alarms. One municipality reported successfully using a written record of the practice and focusing on establishing routines for information flow. The written record was perceived as crucial to ensuring consistent practice across employees in their organisation*.* This resonates with other findings describing how training and information flow created employee confidence. Lack of training and information flow caused a failure to cope, especially for new employees and temporary staff.

In sum, the informants described safe social alarm practice as depending on factors such as the number of care workers available; whether they are visiting a patient; the geography of the municipality; and where the care worker is at the time of the alarm activation as well as the evolution in the social alarm technology.

## Discussion

Our findings revealed that installing, using, and maintaining the social alarm in home care services are basic elements of safety work, and hence safety for home-dwelling patients. Ensuring that the alarms were functioning was a major priority for home care staff. The seemingly trivial work of initiating and maintaining alarm function, was at the same time stressful for staff because the consequences of not immediately attending to a non-functioning alarm could have serious consequences.

Study findings also identified three work processes that care workers engaged in to successfully deliver home care services while contributing to patient/family independence and patient safety via a social alarm. These processes are: Aligning people and technologies, staying alert, and keeping calm, and the coordination of activities that depend on people and technology. We argue that attention to these work processes/elements will contribute to the delivery and organisation of safety work through and with telecare. However, whilst care workers in daily practice already attend to them, the tasks involved often remain invisible to managers, health technology vendors and policymakers.

This study has shown that technology forces new roles and new practices on the care workers to contribute to patient safety using telecare. We also see one of the most crucial aspects of the “technology will replace people workers” debate – someone must assume responsibility and act when the technology triggers the alarm. In the case of the social alarm, this means distinguishing between false alarms and real alarms, which could mean a threat to patient safety.

In addition to the various forms of work performed by the care workers, one should not forget all the work that must be done by patients and families during a caring trajectory. The homecare patients actively participate in shaping the use of the social alarm. The patient receiving care services is inevitably a part of the service, co-creating care with other actors involved. Care workers can offer a service, but it is in the interaction between the care provider and the patient that the care takes place [35]. Furthermore, the integration of technologies tends to result in the delegation of responsibility and work to care workers, the patient and the carers [36]. An example of this in the case of the social alarm is how the users must wear the alarm pendant and activate the alarm when necessary.

From this analysis, we conclude that the idea of technology as an “easy fix” to challenges and risks posed when patients live at home must be subject to scrutiny. This is in line with other literature within the technology in practice literature, such as Pols [9], who have revealed that a lot of tinkering and ethical considerations are part of the relations to telecare on a micro-level. In our study, we have revealed how care workers, patients and next of kin are aware of the complexity of the work to make the social alarm work.

Other researchers have found that technologies that are perceived as easy to understand and use and that have low complexity will be more likely to be accepted and disseminated [37]. Our results support the idea that it is important to make nuanced assessments of goals, intentions, and the individual and community consequences of using the technology in question.

It has been highlighted that implementation of telecare for patients is linked to knowledge of the individual’s preferences and needs, and must be adjusted to these [38], and further that in the development of technical specifications and training, one must ensure that the technology is adapted to the users’ requirements, with a range of opportunities for individual adaptation [39]. In line with the conclusions of Demiris and Shapira [38, 39], our study also indicates that technology competence should be more clearly included in the health and care services and professional health educations, to ensure usability in highly diverse settings and thus foster patient safety across these.

## Conclusions

This paper explored how patient safety in home care is addressed through and with telecare. Based on qualitative data from Norway on the use of social alarms that are widely adopted in home care, we identified three co-existing work processes that are required for the safety alarm to work as intended in contributing to safety for the patients. Home-dwelling patients’ safety is not created by the social alarms on their own but is produced and reproduced through the efforts of care workers vis-à-vis the patient and the technology. Patient safety is the outcome of how humans- the care workers, patients and their family and carers -relate to the devices and to the additional workload entailed in ensuring that these devices function optimally for their patients, including appropriate reactions to alarm signals.

Consequently, we argue that safety work is a particular type of care work that overlaps with other types of work such as comfort work, bodywork, organising work, and machine work. Safety work, like the other work types, is seldom articulated by those involved, yet it requires attention, skills, and knowledge to perform. Furthermore, we claim that the social alarm cannot function on its own, and its implementation can never be finalised. Rather, the social alarm is, and always will be, a continuous everyday process requiring the interaction of care workers, patients, vendors, managers, and policymakers in collaborative ways. Finally, we believe that there is a need to raise awareness of how workers in home care constantly align people and technologies, focus on being alert and staying calm at the same time, and also coordinate activities related to making the social alarm work. This should also be dealt with on a practical level.

To ease the pressure on care workers in an increasingly technology-intensive work context, and to ensure that patient safety is achieved as smoothly as possible when a growing number of welfare technologies are implemented in home care, some aspects need further clarification. Topics that should be discussed include the division of labour on home care shifts, the need for new routines and education in telecare for care workers, and not least: how, and on what level, should decisions on telecare implementation and organisation be made. The alarm management practices include procedures, administrative skills, collaboration with home care recipients and their next of kin, and collaboration between the home care services and their partners, which is crucial to the alarm system infrastructure.

## Data Availability

The empirical data for the current study consisting of interviews and field notes are not publicly available for privacy reasons. The data contain information that could compromise research participant privacy/consent. Consent to share anonymised interview transcripts was not asked for at the time of the research. Requests regarding the data from this study might be directed to corresponding author Randi Stokke.
